# Use of Systems Biology Approaches to Analysis of Genome-Wide Association Studies of Myocardial Infarction and Blood Cholesterol in the Nurses' Health Study and Health Professionals’ Follow-Up Study

**DOI:** 10.1371/journal.pone.0085369

**Published:** 2013-12-26

**Authors:** Dermot Reilly, Ke Hao, Majken K. Jensen, Cynthia J. Girman, Eric B. Rimm

**Affiliations:** 1 Department of Molecular Profiling and Research informatics, Merck Research Laboratories, Merck & Co., Inc., Whitehouse Station, New Jersey, United States of America; 2 Department of Nutrition, Harvard School of Public Health, Boston, Massachusetts, United States of America; 3 Department of Epidemiology, Merck Research Laboratories, Merck & Co., Inc., Whitehouse Station, New Jersey, United States of America; 4 Channing Division of Network Medicine, Department of Epidemiology and Nutrition, Harvard School of Public Health, Boston, Massachusetts, United States of America; 5 Department of Medicine, Brigham and Women's Hospital and Harvard Medical School, Boston, Massachusetts, United States of America; INRCA, Italy

## Abstract

With the advance of genome-wide association studies and newly identified SNP (single-nucleotide polymorphism) associations with complex disease, important discoveries have emerged focusing not only on individual genes but on disease-associated pathways and gene sets. The authors used prospective myocardial infarction case-control studies nested in the Nurses’ Health and Health Professionals Follow-Up Studies to investigate genetic variants associated with myocardial infarction or LDL, HDL, triglycerides, adiponectin and apolipoprotein B (apoB). Using these case-control studies to illustrate an integrative systems biology approach, the authors applied SNP set enrichment analysis to identify gene sets where expression SNPs representing genes from these sets show enrichment in their association with endpoints of interest. The authors also explored an aggregate score approach. While power limited one’s ability to detect significance for association of individual loci with myocardial infarction, the authors found significance for loci associated with LDL, HDL, apoB and triglycerides, replicating previous observations. Applying SNP set enrichment analysis and risk score methods, the authors also found significance for three gene sets and for aggregate scores associated with myocardial infarction as well as for loci-related to cardiovascular risk factors, supporting the use of these methods in practice.

## Introduction

The traditional risk factors for myocardial infarction (MI) include age, plasma lipid concentrations, blood pressure, use of tobacco, and presence of type 2 diabetes mellitus. Family history has also been established as an important risk factor for MI, and is included in some risk scores [1]. While lifestyle clearly plays a role in these risk factors, a large proportion of the inter-individual variability in plasma lipid concentrations is due to inherited factors [2-6]. Premature MI appears to have a particularly strong genetic component independent of established risk factors, and individuals having at least one parent with premature cardiovascular disease (age of onset <55 years in men, <65 years in women) have significantly increased risk of a cardiovascular event [7]. 

These observations have motivated investigators to undertake a variety of studies to identify genes responsible for the heritability of MI and cardiovascular risk factors. For several decades, linkage analyses and candidate gene studies investigated genes responsible for the heritability of cardiovascular risk and successfully identified a handful of loci unequivocally linked to MI, as well as several additional loci with a weaker level of evidence. 

More recently, the emergence of genome-wide association (GWA) arrays have made it possible to perform genome-scale screens for common DNA sequence variants associated with phenotypes of interest including coronary artery disease and MI [8-10]. Genome-wide association studies (GWAS) involving up to 100,000 individuals of European ancestry have identified >90 genetic loci contributing to inter-individual variation in plasma lipid concentrations [11]. Many of these loci harbored genes previously known to influence plasma lipid concentrations. Knowledge of the new sequence variants that confer risk has the potential to illuminate causal biologic pathways in humans and help appropriately target diagnosis and treatment [12]. 

Whereas most prior GWA studies of coronary heart disease (CHD) have been cross-sectional, we performed GWAS using the established prospective MI case-control studies of the Nurses’ Health Study (NHS) and Health Professionals Follow-Up Study (HPFS) cohort to identify common genetic variants that associate with MI, cardiovascular disease biomarkers and lipids. The use of prospective studies avoids survival bias which can confound cross-sectional studies. With integrative analysis approaches, we used expression SNPs (eSNPs), transcriptional networks and expression profiling signatures to explore explanatory relationships between genetic variants identified and disease phenotype. 

## Materials and Methods

### Study Design and Samples

Details of the NHS and HPFS cohorts have been described previously [13,14]. Briefly, the NHS was established in 1976 when 121,700 female registered nurses aged 30–55 years and residing in 11 US states completed a mailed questionnaire on their medical history and lifestyle. The lifestyle factors, including smoking, menopausal status and postmenopausal hormone therapy and body weight, have been updated by validated questionnaires every 2 years. A total of 32,826 women provided blood samples between 1989 and 1990. The HPFS is a prospective cohort study of 51,529 US male health professionals aged 40–75 years at study initiation in 1986. Information about health and disease is assessed biennially by a self-administered questionnaire. Between 1993 and 1999, 18,159 men provided blood samples. Participants from both cohorts underwent local phlebotomy and returned samples to our laboratory via overnight courier. Upon arrival, whole blood samples were centrifuged and stored in cryotubes as plasma, buffy coat, and red blood cells in the vapor phase of liquid nitrogen freezers. DNA was extracted from the buffy coat fraction of centrifuged blood with the QLAmp Blood Kit (Qiagen, Chatsworth, California). 

NHS and HPFS participants for the current study were those with a blood sample in the nested case–control studies of incident CHD, defined as non-fatal MI or fatal CHD. In the NHS, 474 women free of cardiovascular disease or cancer in 1990 and 454 men in HPFS free of cardiovascular disease in 1994 developed incident CHD prior to June 2004. For each incident case of CHD, 2 men or women who were free of cardiovascular disease were randomly selected matching on age (in 5-year increments), smoking (in 5 categories), and month of blood return using risk-set sampling [15]. In the NHS, matching criteria also included fasting status and reported problems with blood sampling. 

### Ethics Statement

The present study was approved by the institutional review board of the Brigham and Women's Hospital and the Human Subjects Committee Review Board of Harvard School of Public Health. All documents sent to participants have been approved by the ethics committee.

We wrote to participants who reported incident CHD on the follow-up questionnaires to confirm the report and request permission to review medical records. Participants, or the next of kin in the case of deceased participants, provided implied written consent by the return of the mailed questionnaires. However, further consent to review medical records was through signed written consent. To document the process, upon reporting an incident MI, participants were recontacted by mail to confirm the report and to obtain written consent to review medical records. We also sought medical records for deceased participants, whose deaths were identified by families and postal officials and through the National Death Index. Physicians blinded to the participant’s questionnaire reports reviewed all medical records. Cases were identified primarily through review of medical records, as previously described [14,16]. 

### Genotyping and Quality Control

Genotyping was done using the Affymetrix SNP 6.0 array and the Birdseed calling algorithm [17]. Genotypic data for a total of 1330 HPFS samples (98%) passed laboratory technical quality control criteria. Likewise, 96% of the NHS samples were successfully genotyped. A subset of 312 NHS samples were not genotyped together with the remaining CHD case-control set as they overlapped with previous GWA studies of breast cancer (Illumina 550) and type 2 diabetes (Affymetrix 6.0). These samples were quality controlled as part of the earlier GWAS (leaving n=272 samples with available data) and SNPs, also present on the Affymetrix 6.0 platform, were subsequently merged with the CHD data, after removing samples with the following criteria: 1) accidental duplicates as identified by pairwise identity-by descent; 2) samples identified as siblings; 3) discordant genotypic and phenotypic sex; and 4) sample call rate <98%. Population structure was investigated by principal component analysis. We used a set of 12,021 SNPs selected to have very low levels of linkage disequilibrium and minor allele frequency >0.05 in Caucasians [18]. Study subjects passing quality control were analyzed together with a set of 209 HapMap II founders (59 CEU, 60 YRI, 45 JPT and 45 CHB). Subjects within the means of the first and second principal components [mean (SD) = 3] among self-described Caucasians were classified as having primarily European ancestry. Due to very few samples with substantial evidence of non-European genetic ancestry, these samples were excluded from subsequent analysis (n=24). SNPs that were monomorphic, had a missing call rate ≥2%, a Hardy-Weinberg Equilibrium p-value <1×10-4, or a minor allele frequency <0.02 were excluded, leaving a total of 724,881 SNPs that passed quality control in HPFS and 721,316 in NHS for analysis of called genotypes. Imputation of ~2.5 million SNPs was performed using MACH software (v1.0.16) with HapMap CEU phased II data (Release 22) as the reference panel.

### Statistical Analysis

We performed unconditional logistic regression to analyze the association between each assayed or imputed SNP and incident CHD risk using an additive genetic model. Unconditional regression was used because some of the original matches were broken when informed consent had to be obtained a second time. NHS and HPFS were examined separately. The genomic inflation factor was adjusted by genomic control methods. To control for potential confounding by population stratification, we performed further analyses by including the top principal components of genetic variation chosen for each study in the models. Adjusting for the top three and four principal components for NHS and HPFS, respectively, made no material difference to the GWA results.

We used two meta-analytic methods to summarize the statistical evidence for each SNP. We combined odds ratios for a given reference allele on a logarithmic scale weighted by the inverse of their variances using a fixed-effects model. We also combined evidence for association solely on the basis of signed p-values weighted by sample size. In brief, we converted the two-sided p-value to a z-statistic and assigned a sign to reflect the direction of the association given the reference allele. Each z-score was then weighted with the squared weights summing to 1, with sample-specific weights being proportional to the square root of the effective number of individuals in the sample. 

All analyses were implemented using R (version 2.12).

### SSEA

**Figure 1:**
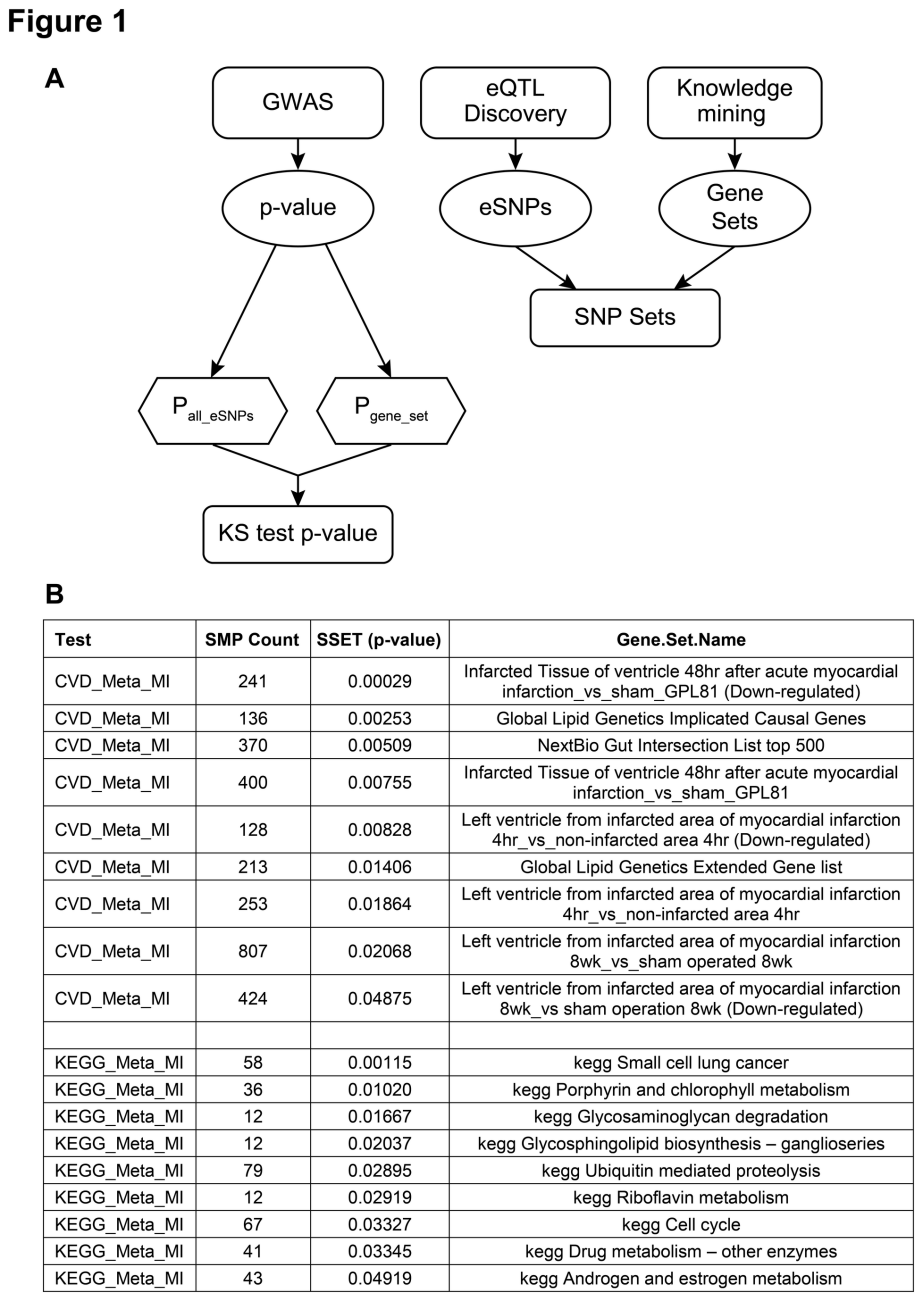
SNP set enrichment test work flow. **A**: 100 gene sets derived from mRNA profiling signatures, gene expression networks and other sources relevant to dyslipidemia and cardiovascular disease were assembled. Each gene set was sequentially tested against the associations identified for the phenotype of interest. For each gene set, multiple eSNPs representing the genes in that gene set were tested as a group for association to the trait of interest using a Kolmogorov-Smirnov test. **B**: Gene sets corresponding to Kegg pathways were also tested for the phenotype of MI.

A SNP set enrichment (SSEA) test [19,20] was applied in an attempt to identify underlying biological signals within the many suggestive associations observed for MI in this dataset. Briefly, more than 100 gene sets derived from mRNA profiling signatures, gene expression networks and other sources relevant to dyslipidemia and cardiovascular disease were assembled (Table S1 and Table S2). Each gene set was sequentially tested against the associations identified for the phenotype of interest (Figure 1). For each gene set, multiple eSNPs representing the genes in that gene set were tested as a group for association to the trait of interest using a Kolmogorov-Smirnov test. Gene sets corresponding to Kegg pathways were also tested for the phenotype of MI. 

### Testing for Gene Environment Interaction

Taking advantage of the rich environmental data captured in this cohort, we examined gene-environment interactions for HDL, apoB, LDL, smoking, and alcohol in relation to incident MI using both linear and non-linear terms. Given the additional tests, although not independent, a conservative Bonferroni threshold of p<1E-9 was considered for genome wide significance We also constructed a two degree of freedom test for main and interaction effects in the context of nested models. The test is essentially equivalent to that proposed by Kraft et al. [21]. By comparing a model with both main effects and the interaction between the gene and the environmental factor to one with only the main effects, a likelihood ratio test was formed to screen (1) SNPs which may have significant main or interaction effects, and (2) SNPs may only show significant associations when interaction is considered. Both of these models were fit using the continuous linear and non-linear variable for the trait of interest (alcohol, HDL, total cholesterol, apoB). We constructed four sets of logit models, and tested the SNP by environmental interactions by comparing the full model vs the null model in each set after adjusting for age, smoking and principal components. The four models differed by whether the environmental factor was fit using a polynomial spline function with three degrees of freedom or a continuous linear function (methods S1). Finally, we reported significant interactions in any of those models. 

### Predicting MI Risk using SNPs Underlying Lipids and Other Risk Factors

A genotype risk score similar to that of Purcell et al. [22] was applied to evaluate whether common genetic variants have an important role in the prediction of MI risk. Using increasing thresholds for the number of SNPs included (based on ascending p-value), we defined large sets of ‘score alleles’ from the cardiovascular biomarkers [LDL, HDL, apoB, adiponectin, c-reactive protein (CRP), triglycerides, total cholesterol] and the MI associated loci to generate aggregate risk scores. In brief (1), we selected the top N SNPs from each GWA study on a given lipid trait, avoiding SNPs in strong linkage disequilibrium (LD) by forcing the SNPs to be at least 100 kb apart (2). For each subject we compiled a risk score based on his/her genotypes of the selected SNPs. For a SNP associated with LDL, total cholesterol, apoB or triglycerides, if the subject was homozygous for a SNP linked to higher lipid level, we added "+1" to his/her risk score; if homozygous for a SNP linked to lower lipid level, we added "-1"; a heterozygous genotype was assigned "0". Signs were reversed for HDL and adiponectin in the risk score (3). The association of each subject's risk score to MI was tested in a logistic regression model and significant results indicated prediction power to MI.

## Results

### Genome-wide Association for MI and Blood Markers

Cases were matched to controls on age but cases but had a greater prevalence of hypertension, diabetes and smoking (Table 1). No loci reached genome-wide thresholds for statistical significance for MI. Several SNP loci reached genome wide significance (Table 2) for apoB (rs1713222; gene: *APOB*; rs445925; *APOE*, *APOC1*), LDL cholesterol (rs445925; *APOE*, *APOC1*), HDL cholesterol (rs3741298; *APOA5*, *APOA4*, *APOC3*; rs1532625; *CETP*) and triglycerides (rs964184; *ZNF259*, *APOA5*, *APOA4*). Notable borderline loci observed for apolipoprotein B (Table 3) included (rs17765901; *MSRA*) and for LDL included (rs563290; *APOB*) and (rs12579637; *TMEM16D*) which was also a suggestive locus identified for apoB levels. 

**Table 1:** Clinical parameters for the case control cohorts in the NHS and HPFS.

	**HPFS**		**NHS**	
**Characteristic**	**Cases**	**Controls**	**Cases**	**Controls**
N	425	878	464	945
Age, years	64.5 (8.6)	64.2 (8.5)	60.2 (6.3)	59.8 (6.3)
Women, %	0%	0%	100%	100%
Hypertension,^†^ %	37.20%	29.00%	50.20%	27.30%
Diabetes,^†^ %	9.00%	3.80%	14.40%	6.24%
Current smoker, %	9.70%	8.70%	27.80%	26.10%
Total cholesterol, mmoL	5.5 (1.0)	5.2 (1.0)	6.1 (1.0)	5.9 (1.0)
HDL cholesterol, mmoL	1.1 (0.3)	1.2 (0.3)	1.3 (0.4)	1.6 (0.4)
Triglyceride, mmoL	1.8 (1.5)	1.5 (2.2)	1.6 (1.0)	1.3 (0.7)
BMI, kg/m^2^	26.0 (3.2)	25.6 (3.3)	26.0 (6.6)	24.5 (5.8)

Abbreviations: BMI: Body Mass Index; HPFS: Health Professionals follow up study; NHS: Nurses’ Health Study; N: number of individuals.

^†^ Diabetes and hypertension were based on self-reports of physician diagnoses.

**Table 2:** SNP associations exceeding thresholds for genome-wide significance.

**Trait**	**Snp**	**Allele**	**Maf**	**Location**	**z1(NHS)**	**z2(HPFS)**	**Z(Meta)**	**P value**	**Gene**
ApoB	rs1713222	G A	0.17	2p24.1	-2.8	-4.8	-5.5	4.25E-08	ApoB
ApoB	rs445925	A G	0.14	19q13.32	3.0	4.8	5.6	2.00E-08	ApoE, ApoC1
LDL	rs445925	A G	0.14	19q13.32	5.5	4.8	7.1	1.10E-12	ApoE, ApoC1
HDL	rs3741298	A G	0.20	11q23.3	-4.5	-4.2	-6.1	8.48E-10	ApoA5, ApoA4, ApoC3
HDL	rs1532625	A G	0.44	16q13	-6.0	-5.2	-7.8	6.00E-15	Cetp
Triglycerides	rs964184	C G	0.13	11q23.3	4.6	5.4	7.0	2.87E-12	Znf259, ApoA5, ApoA4
Adiponectin	rs822387	C T	0.09	3q27.3	-6.3	-0.6	-6.0	1.71E-09	Adipoq
CRP	rs2027471	T A	0.34	1q23.2	-4.5	-5.0	-6.7	1.80E-11	Crp
CRP	rs440638	A G	0.18	19q13.32	-3.9	-7.2	-8.1	4.44E-16	ApoC1, ApoE

Abbreviations: Allele, Risk Allele & reference allele; ApoB: Apolipoprotein B; CRP: C-reactive protein; location, chromosome location; Gene: nearest gene(s) to associated SNP; HPFS: health professionals follow- up study; HDL: high density lipoprotein; LDL: low density lipoprotein; MAF, minor allele frequency; Meta: meta-analysis; NHS: nurses’ health study; rs, Reference SNP; SNP, single nucleotide polymorphism; z1/z2/Z: z-score from individual cohorts and meta-analysis

The p value threshold for genome wide significance in this study was 5x10E-8.

**Table 3:** Suggestive SNP associations for traits of interest.

**Trait**	**Snp**	**Allele**	**Maf**	**Location**	**z1(NHS)**	**z2(HPFS)**	**Z(Meta)**	**P value**	**Gene**
Myocardial Infraction	rs10954357	C T	0.28	7q32.3	3.4	3.2	4.7	2.66E-06	desert, Podxl, Plxna4b
Myocardial Infraction	rs7497692	C T	0.22	15q14	3.6	3.0	4.7	3.28E-06	Ryr3
ApoB	rs17765901	G T	0.48	8p23.1	1.7	4.7	5.0	6.93E-07	Msra
ApoB	rs1347992	A G	0.23	2q24.3	-1.7	-4.6	-4.9	8.90E-07	Scn3a
ApoB	rs11110561	C A	0.34	12q23.1	-2.1	-4.3	-4.8	1.62E-06	Tmem16d
ApoB	rs3102078	C A	0.35	8p23.1	3.2	3.7	4.8	1.75E-06	Ppp1r3b
ApoB	rs10201348	G A	0.14	2q31.2	-2.0	-4.3	-4.7	2.26E-06	Znf533
ApoB	rs11569377	T C	0.07	12p13.31	2.3	4.1	4.7	2.50E-06	Cd27
ApoB	rs4974961	C T	0.38	4p14	2.1	4.2	4.7	2.81E-06	N4bp2
ApoB	rs4915789	A G	0.36	1p31.3	-2.9	-3.8	-4.6	3.33E-06	Inadl
ApoB	rs2423066	G A	0.25	20p13	2.4	4.0	4.6	3.42E-06	Slc23a2, Rassf2
ApoB	rs11036434	A G	0.33	11p12	-1.4	-4.4	-4.6	4.39E-06	Lrrc4c, Api5
ApoB	rs4688296	A T	0.46	3p14.2	-2.9	-3.7	-4.6	4.44E-06	Cadps
HDL	rs698739	G T	0.14	8q23.3	-3.5	-3.4	-4.9	1.09E-06	desert, Eif3s3, C8orf53
HDL	rs12998922	G C	0.23	2q37.1	-2.2	-4.5	-4.9	1.17E-06	Spp2, Arl4c, Trpm8
HDL	rs1581675	A T	0.27	8p21.3	-3.1	-3.6	-4.8	1.55E-06	Lpl
HDL	rs13279426	A G	0.11	8p23.2	-4.4	-2.6	-4.8	1.63E-06	Csmd1
HDL	rs11635491	A G	0.29	15q22.1	-3.4	-3.4	-4.8	1.78E-06	Lipc
HDL	rs10271980	A G	0.40	7q11.22	-2.1	-4.4	-4.8	1.96E-06	desert, Auts2
HDL	rs17112078	A G	0.35	14q32.33	3.1	3.6	4.7	2.58E-06	desert, Tmem121, Crip1, Crip2
HDL	rs1570513	A G	0.26	9p24.1	-2.9	-3.6	-4.6	4.51E-06	Jmjd2c
LDL	rs10963243	A G	0.07	9p22.2	3.6	3.8	5.1	3.20E-07	Sh3gl2
LDL	rs563290	A G	0.19	2p24.1	-1.6	-4.5	-4.6	3.43E-06	ApoB
LDL	rs711873	G T	0.20	11q13.4	2.6	3.8	4.6	3.89E-06	Slc02b1, Arrb1
LDL	rs12579637	A G	0.23	12q23.1	-1.9	-4.2	-4.6	4.39E-06	Tmem16d
TG	rs6993414	G A	0.11	8p21.3	4.3	3.0	5.2	2.14E-07	Lpl
TG	rs780094	A G	0.40	2p23.3	-4.1	-3.2	-5.1	2.93E-07	Gckr
TG	rs6583343	C G	0.45	7p22.3	3.4	3.4	4.8	1.77E-06	Fam20c
TG	rs9385770	C T	0.35	6q23.3	-3.8	-3.0	-4.8	1.85E-06	Map3k5
TG	rs7656846	A C	0.31	4q25	3.1	3.6	4.7	2.35E-06	Papss1
TG	rs9860363	T C	0.07	3q26.1	-3.9	-2.5	-4.6	3.47E-06	desert, Bche
TG	rs437895	A G	0.40	8p23.1	3.8	2.6	4.6	4.68E-06	Mfhas1, Cldn23, Thex1
Adiponectin	rs8006060	G A	0.14	14q22.1	-4.6	-1.9	-5.0	6.50E-07	Ptgdr, Ptger2
Adiponectin	rs6862233	A G	0.28	5q14.1	-4.6	-1.2	-4.7	2.24E-06	Serin5c, Thbs4
Adiponectin	rs1606328	C G	0.31	11q22.3	4.2	2.2	4.7	3.14E-06	desert, Gucy1a2, Aasdhppt
Adiponectin	rs1514989	C T	0.43	4p15.32	4.3	1.7	4.6	3.57E-06	Ldb2
Adiponectin	rs6437640	C G	0.18	3q13.11	-4.3	-1.5	-4.6	4.04E-06	Cblb
Adiponectin	rs2270294	G T	0.12	7p14.1	-4.0	-2.5	-4.6	4.50E-06	Amph
CRP	rs7979473	G A	0.39	12q24.31	-4.7	-2.9	-5.3	1.08E-07	Tcf1(Hnf1a),C12orf43

Abbreviations: Allele: Risk Allele & reference allele; ApoB: Apolipoprotein B; CRP: C-reactive protein; Gene: nearest gene(s) to associated SNP; HDL: high density lipoprotein; HPFS: health professionals follow-up study; LDL: low density lipoprotein; location: chromosome location; MAF: minor allele frequency; Meta: meta-analysis of both cohorts; NHS: Nurses’ health study; rs: reference snp; SNP: single nucleotide polymorphism; z1/z2: meta-analysis z-score; z1/z2/Z: z-score from individual cohorts and meta- analysis

The p value threshold for genome wide significance in this study was 5x10E-8.

Loci with suggestive associations for HDL included (rs1581675, *LPL*) and (rs11635491; *LIPC*). Suggestive loci identified for triglycerides include 8p21 (rs6993414, near *LPL*), 2p23 (rs780094, near *GCKR*) and 4q25 (rs7656846, near *PAPSS1*).

SNP loci reaching thresholds for genome wide significance (Table 2) were observed for adiponectin (rs822387, *ADIPOQ*), and CRP (rs2027471; *CRP* and rs4420638; 19q13.32, *APOC1*, *APOE*). No significant loci were identified for creatinine. Suggestive associations observed for adiponectin levels included 14q22 (rs8006060, *PTGDR*, *PTGER2*) which are prostaglandin receptors for *PGD2* and *PGE1* respectively. Suggestive associations for CRP included the 12q24 locus (rs7979473; *HNF1α*). 

### SNP Set Analysis for MI and apoB

**Figure 2:**
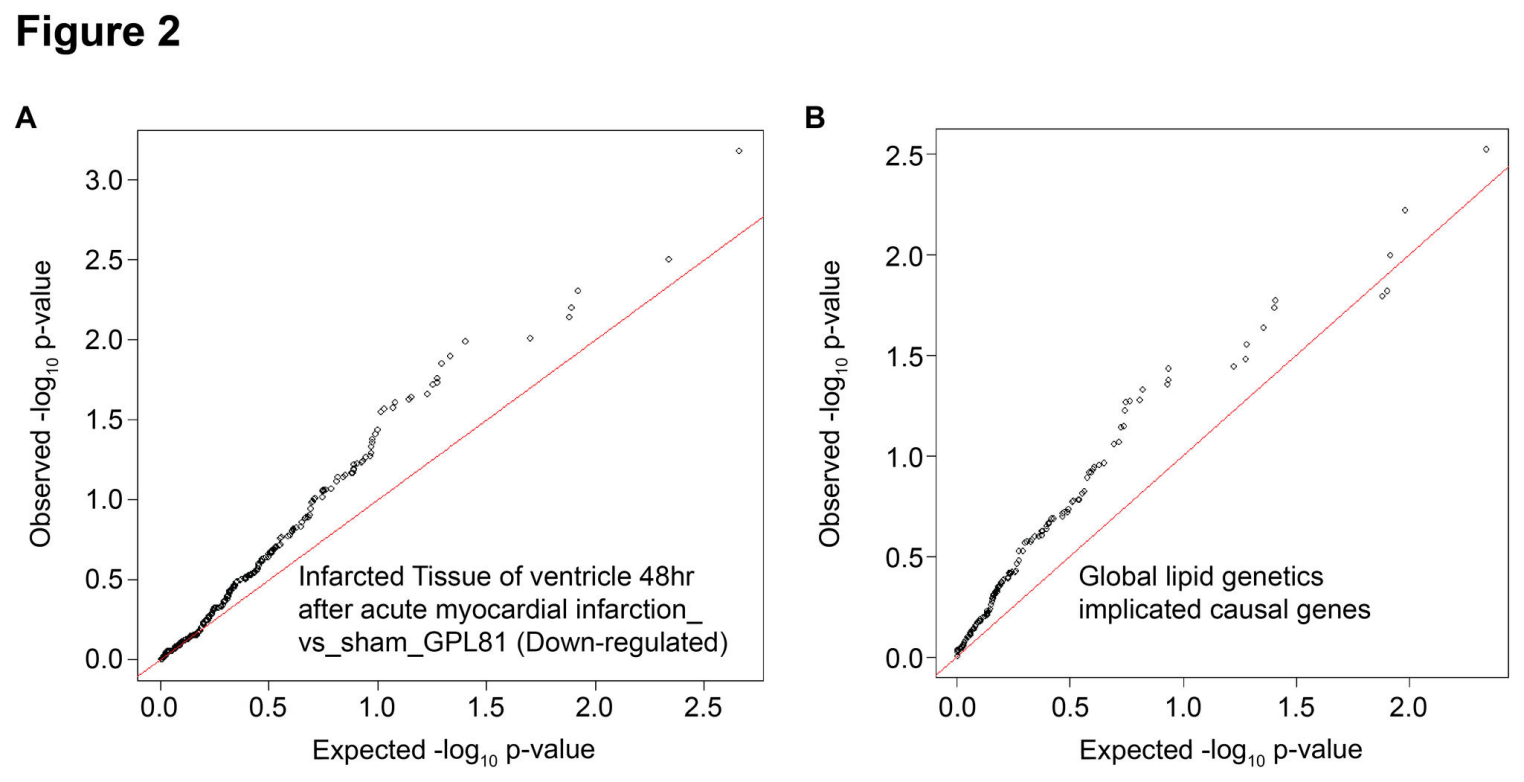
Quantile-Quantile(QQ) plots on SNP set enrichment. QQ-plots of SNP set enrichment test. Solid line represents normal distribution and black dots represent data distribution of p-values for eSNPs representing genes within the gene set of interest. Deviation of the data from the solid line indicates a non-normal distribution. Plots are shown for the top ranking gene sets A: published mRNA profiling signatures from infarcted tissue and B: implicated causal genes from the Global Lipid Genetics GWAS Consortium.

In SSEA analysis, the top gene sets associated with MI (meta-analysis) included published mRNA profiling signatures from infarcted tissue (p=2.9E-4), implicated causal genes from the Global Lipid Genetics GWAS Consortia (p=2.5E-3) (Figure 2), and a gene set formed from the intersection of many gut profiling signatures in the Nextbio database (p=5.0E-3). Kegg pathway gene sets significantly associated with MI included small cell lung cancer (p=1.1E-3), porphyrin and chlorophyll metabolism (p=1E-2) and glycosaminoglycan degradation (p=1.6E-2). Finally, for the 29 MI loci previously identified and replicated by the Myocardial Infarction Genetics, Cardiogram or the C4D Consortia [9,10,23] no statistically significant individual SNPs or gene sets were observed. 

### Gene Environment Interactions Examined for Myocardial Infarction

SNP interactions that reached or approached genome-wide levels of significance included rs11147396 with alcohol for MI; rs3806850 and HDL for MI; rs17542348 with cholesterol for MI in HPFS only; and rs10794792 with apoB for MI (Table 4). None of these associations showed a significant association to MI when examined for main effect in the meta-analysis or in HPFS only. SNP rs17542348 was marginally associated with MI risk (p value=3.7E-6) in NHS, where T was the risk allele. 

**Table 4:** Gene: environment and gene: intermediate interactions with incident MI reaching genome-wide significance.

**Intxn**	**Chr**	**rsID**	**Study**	**Model 1**	**Model 2**	**Model 3**	**Model 4**	**Near Gene**	**MI Main Effect p**
Alcohol : MI	13q12.3	rs11147396	meta	8.1E-02	1.9E-01	**3.0E-09**	**1.7E-08**	Ubl3, Slc7a1	6.7E-01
HDL : MI	5p13.3	rs3806850	meta	**2.0E-09**	**2.6E-08**	9.4E-07	3.2E-06	Cdh6	4.3E-01
Chol : MI	7p21.3	rs17542348	HPFS	3.1E-05	**2.5E-09**	6.6E-04	**5.2E-08**	Phf14	3.2E-01
ApoB : MI	10p15.3	rs10794792	meta	1.5E-07	3.2E-06	**1.3E-08**	1.2E-07	Adarb2 (Idl1, Idl2)	9.4E-01

Abbreviations: Chr: chromosome; Intxn: interaction examined; MI: Main Effect; Model: statistical model utilized; Near Gene: nearest gene(s) to associated SNP; p: association p-value for main effect analysis of Myocardial Infarction; rsID: reference snp identifier; Study: study population meta-analysis of distinct cohort (NHS, HPFS)

^a^ Models 1-4 defined in supporting information (Text S1).

### Genotype Risk Score of Cardiovascular Risk Factors and the Prediction of MI

Not surprisingly, aggregate risk scores derived from biomarker meta-analysis associations (LDL, HDL, apoB, adiponectin, CRP) associated strongly with MI in both the HPFS and NHS cohorts individually, with increasing significance level as the number of SNPs in the score increased. Scores derived from biomarker associations in HPFS or NHS did not replicate when tested across gender, and scores from MI associations did not show strong prediction across gender (HPFS derived associations tested in NHS). When scores were derived based on associations from both MI and biomarker associations analyses in either HPFS or NHS, moderate levels of association were found for the prediction of MI across gender with increasing levels of significance observed as the number of SNPs included in the score increased. Testing of these risk score paradigms did not replicate in the Wellcome Trust Case Control Consortium GWAS for CHD. 

## Discussion

MI is a leading cause of death and disability worldwide, and the inherited basis for myocardial infarction remains incompletely understood, although substantial progress has been made recently through the application of GWAS [9,10,23–25]. Applying GWAS to our prospective case-control studies of MI, we found no loci reaching genome wide significance levels, and no association signals at 9p21.3 which has previously been associated with early onset myocardial infarction or CAD [9,24,25]. Restricting the age of MI to younger individuals did not elucidate an association signal around 9p21, although power was even more limited. Similarly, no strong associations were observed at up to twenty nine other MI loci previously identified and replicated by the Myocardial Infarction Genetics, Cardiogram or the C4D Consortia [9,10,23]. We believe the absence of apparent association between genetic variants and MI is likely due to limited sample size. After this study was initiated, it became apparent the current cohort was substantially underpowered for the primary endpoint of MI. It is also possible that the prospective study design influenced the likelihood of detecting loci discovered predominantly in cross sectional studies. However larger prospective cohorts will be required to evaluate this possibility. 

 Significant SNP associations with blood cholesterol phenotypes LDL, HDL, apoB and triglycerides were observed, successfully replicating numerous previous observations [11,26]. The identification of the *APOB* locus as the one most significantly associated with circulating apoB levels confirms that meaningful information can be derived from GWAS with quantitative traits using sample sizes in this range. Similarly, SNPs in close proximity to the gene encoding the C-reactive protein exhibited the strong associations with CRP levels. 

Notable suggestive loci observed for apoB have been previously associated with adiposity [27] and hypertension [28]. The *MSRA* locus contains eSNPs for *MSRA* (MGH subq), SOX7 (MGH Liver, deLiver) and others [29]. The suggestive locus around *SCN3A* is also supported by an expressional SNP for *SCN3A* in subcutaneous adipose tissue. FXR (Nr1h4) is proximal to the *TMEM16D* locus identified. One of the primary functions of FXR activation is the suppression of cholesterol 7 alpha-hydroxylase (CYP7A1), the rate-limiting enzyme in bile acid synthesis from cholesterol. The *N4BP2* locus also contains a (MGH) Liver eSNP to *N4BP2* but no evident biological rationale. 

In the context of complex human conditions, the effect size for a given causal SNP is often very small. Therefore very large sample size (e.g. N>10,000) would be required to appropriately power detection of such SNPs. An alternative approach is to use the SNP Set Enrichment Analysis (SSEA) to determine whether a group of SNPs, identified by association with gene sets, is enriched for small p-values in GWAS results by examining the effect of many SNPs, as a group, simultaneously. SSEA gains statistical power by (1) reducing the number of tests and (2) pooling genetic effects from multiple weak potentially causal SNPs. Many Kegg and biologically relevant gene sets were assembled and tested independently, and while the individual SNP p-values were moderate, as a set, we observed globally significant SSEA p-values. The top gene sets associated with MI (meta-analysis) included published mRNA profiling signatures from infarcted tissue [30], implicated causal genes from the Global Lipid Genetics Consortia GWAS [11], and a gene set formed from the intersection of many gut mRNA profiling signatures in the Nextbio database (www.nextbio.com). The gene signatures from infarcted tissue were derived in a mouse model of MI and it is striking that the expression quantitative trait loci for such a gene set would show a significant association with MI when examined together. Similarly, expression quantitative trait loci for genes underlying the Global Lipid Genetics Consortia GWAS loci show a significant association with MI. This is perhaps not that surprising given the established relationship in MI prediction for many of the LDL loci in particular. The gut mRNA profiling signatures were chosen predominantly on their relevance to cholesterol related experiments so this may be reflected in the association identified. Three Kegg pathway gene sets were significantly associated with MI, but no evident biological rationale exists for these Kegg pathways. Previously reported MI GWAS loci only achieve moderate p-values in our cohorts (partially due to sample size and statistical power). 

A number of SNP-environment interactions were observed that reached or approached genome wide levels of significance although none of these SNPs were significantly associated with MI as main effects. The interaction of a locus on 13q12 with alcohol has not been previously reported in association with MI. No biological rationale is evident however, for the genes Ubl3 or SLC7A1 that lie in the region of the SNP association. The gene for Cadherin 6 underlies the interaction observed at 5p13 for HDL in association with MI. Cadherins mediate cell-cell binding and contribute to the sorting of heterogeneous cell types and the maintenance of orderly structures in tissue, but no role has yet been described in lipoprotein metabolism. An interaction for total cholesterol and a SNP at 7p21 was observed for MI in the HPFS cohort only. This SNP is near Phf14, a nuclear phosphoprotein that may function as a colon cancer tumor suppressor. Finally, an interaction between apoB and rs10794792 for MI was identified at 10p15 near the gene location for *ADARB2*. *ADARB2* encodes Adenosine deaminase RNA-specific B2, which binds to single-stranded and double-stranded RNAs, and may play a role in regulation of substrate-specific RNA editing. Hepatic apoB mRNA editing determines the proportion of VLDL that contains full length (apoB100) or truncated (apoB48) RNA; however, *ADARB2* is not known to play a role in this process. Additional replication studies are warranted for these interactions in independent cohorts. 

## Conclusions

We used prospective case-control studies of MI to illustrate application of novel SSEA methods and a risk score approach as a means of assessing groups of loci that may be predictive of MI, despite marginal individual loci effects. While power limited our ability to detect significance for individual loci with MI, we found significance for loci associated with LDL, HDL, apoB and triglycerides, replicating previous observations. Applying SSEA and risk score methods, we found significance for three gene sets and for aggregate scores associated with MI as well as for loci related to cardiovascular risk factors, supporting the utility of these methods in practice. 

## Supporting Information

Table S1 **HPFS Gene Sets MI.**
(DOCX)

Table S2 **NHS Gene Sets MI.**
(DOCX)

Text S1 **Supplemental Methods.**
(DOCX)
