# Supplementary figures and images for: Use of Systems Biology Approaches to Analysis of Genome-Wide Association Studies of Myocardial Infarction and Blood Cholesterol in the Nurses' Health Study and Health Professionals’ Follow-Up Study
Source: PLoS One. 2013 Dec 26;8(12):e85369. doi: 10.1371/journal.pone.0085369 (PMC3873433; doi:10.1371/journal.pone.0085369)

**Table S1 HPFS Gene Sets MI**


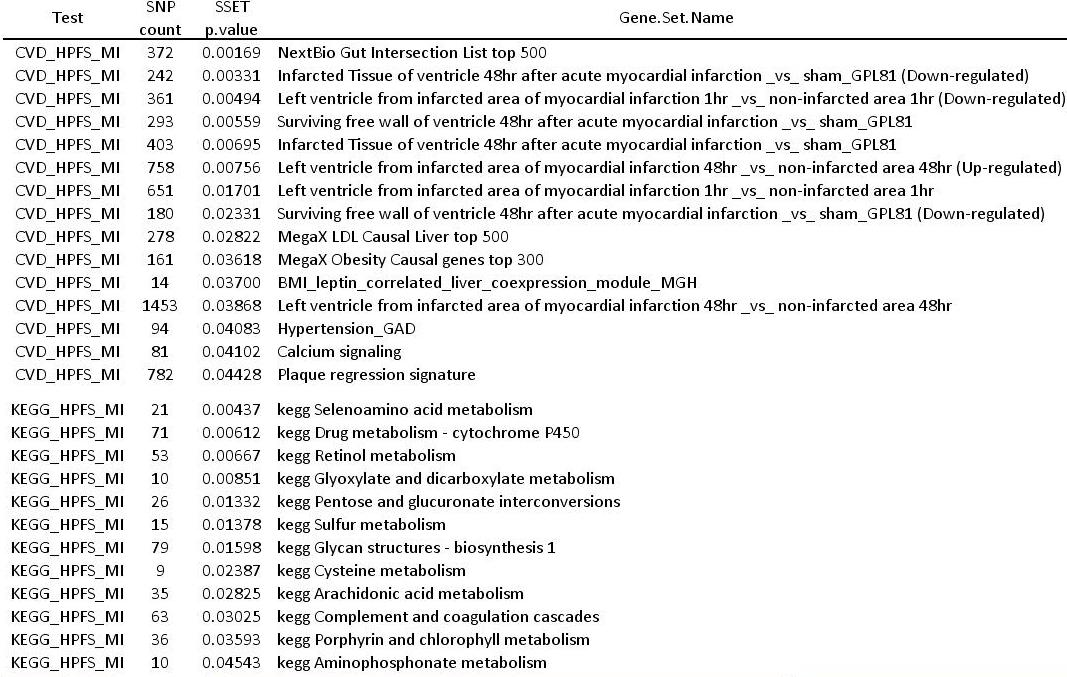

Supplement: Table S1 — HPFS Gene Sets MI. (DOCX) [file pone.0085369.s001.docx]

**Table S2 NHS Gene Sets MI**


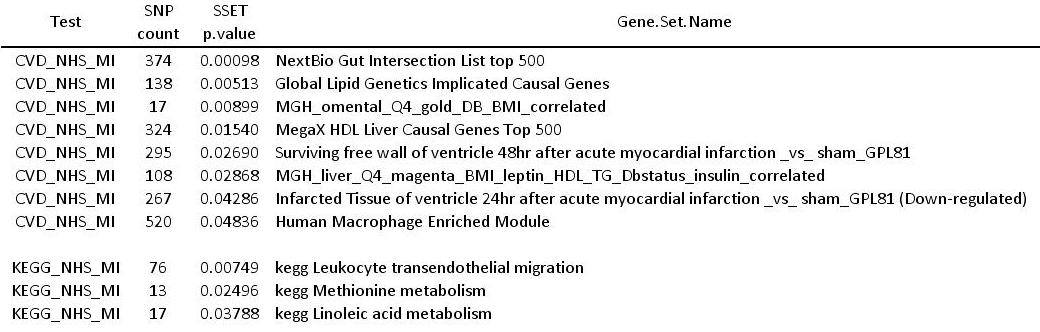

Supplement: Table S2 — NHS Gene Sets MI. (DOCX) [file pone.0085369.s002.docx]
