# Supplementary material for: Use of Systems Biology Approaches to Analysis of Genome-Wide Association Studies of Myocardial Infarction and Blood Cholesterol in the Nurses' Health Study and Health Professionals’ Follow-Up Study
Source: PLoS One. 2013 Dec 26;8(12):e85369. doi: 10.1371/journal.pone.0085369 (PMC3873433; doi:10.1371/journal.pone.0085369)
Supplement: Text S1 — Supplemental Methods. (DOCX) [file pone.0085369.s003.docx]

**Text S1 Supplemental Methods**

**Testing for gene environment interaction**

We constructed a two degree of freedom test for main and interaction effects in the context of nested models. The test detects SNPs with main effects and SNPs with heterogeneity in genetic effects across different levels of the environmental variable. The test is essentially equivalent to that proposed by Kraft et al. (Kraft et al., 2007). In brief, the null model is stated as

E(Y|E) = β_0,M_ + β_1,M_ E (1) ,

where M indicates coefficients of the main effect model, E is the environment variable. Further, the full model is specified as

E(Y|E,SNP) = β_0,I_ + β_1,I_ E + β_2,I_ SNP+ β_3,I_ E × SNP (2) ,

where I indicates coefficients of the interaction model, and SNP is coded as the number of the effective allele.

By comparing these two models, a likelihood ratio test can be formed to screen (1) SNPs which may have significant main or interaction effects, and (2) SNPs may only show significant associations when interaction is considered. Both of these models were fit for interactions of interest using the continuous linear and non-linear (spline) variable for the trait of interest (alcohol, HDL, cholesterol, apolipoprotein b).

Model (Set) 1,

Full Model, logit(MI)~ age + smoking + eigenvectors + genotype + env + genotype:env

Null Model, logit(MI)~ age + smoking + eigenvectors + genotype + env

Model (Set) 2,

Full Model, logit(MI)~ age + smoking + eigenvectors + genotype + env + genotype:env

Null Model, logit(MI)~ age + smoking + eigenvectors + env

Model (Set) 3,

Full Model, logit(MI)~ age + smoking + eigenvectors + genotype + poly(env,3) + genotype:poly(env,3)

Null Model, logit(MI)~ age + smoking + eigenvectors + genotype + poly(env,3)

Model (Set) 4,

Full Model, logit(MI)~ age + smoking + eigenvectors + genotype + poly(env,3) + genotype:poly(env,3)

Null Model, logit(MI)~ age + smoking + eigenvectors + poly(env,3)

In the interaction models, env denoted environmental exposure (e.g. alcohol) and poly(env, 3) denote polynomial spline of three degree of freedom.
